# Exploratory Evaluation of the Fibrosis-4 index, Albumin-bilirubin score, and Neutrophil-lymphocyte ratio (FAN) Sepsis Score for Prognosis in Patients With Poor Performance Status and Sepsis Due to Obstruction by Urinary Calculi

**DOI:** 10.7759/cureus.96169

**Published:** 2025-11-05

**Authors:** Sohei Iwagami, Masaya Nishihata, Haruka Miyai

**Affiliations:** 1 Urology, Kishiwada Tokushukai Hospital, Kishiwada, JPN

**Keywords:** frailty, scores, sepsis, sepsis shocks, urinary tract stone

## Abstract

Background/Objectives: This study aimed to exploratory evaluate the association between fibrosis-4 index, albumin-bilirubin score, and neutrophil-lymphocyte ratio (FAN) sepsis (FANs) score, a sepsis score derived from fibrosis-4 index, albumin-bilirubin score, and neutrophil-lymphocyte ratio, and the prognosis of patients with poor performance status (PS) and sepsis associated with urinary tract stone obstruction.

Methods: We identified 56 patients with PS of 3 or 4 who were admitted to Kishiwada Tokushukai Hospital between April 2019 and March 2025 for septic shock due to obstructive pyelonephritis caused by a urinary tract stone. Patient information was collected retrospectively, and the association between prognosis and FANs score was evaluated.

Results: Patient characteristics were evaluated separately for each score group. Although differences were observed in the use of vasopressors, mean arterial pressure, procalcitonin, platelet counts, C-reactive protein, serum creatinine, and sequential organ failure assessment (SOFA) scores (p=0.04, p=0.01, p<0.01, p<0.01, p=0.01, p<0.01, and p<0.01, respectively), there were no other significant differences between the two groups. An increase in the FANs score was associated with increased mortality (p<0.01). Receiver operating characteristic (ROC) curve analysis showed that the FANs score had predictive ability equivalent to the SOFA score for in-hospital, one-year, and overall mortality.

Conclusions: The FANs score may be associated with the prognosis of patients with poor PS and sepsis due to urinary tract stones.

## Introduction

Sepsis remains a leading cause of death worldwide, particularly among older adult patients with poor performance status (PS) [1-5]. Many of these patients continue to face high mortality rates in both the medium and long term [6,7]. Urinary tract infections (UTIs) are one of the leading causes of sepsis, and UTIs due to stone formation are increasing in the older adult population. Poor PS is associated with poor prognosis and increased perioperative risks; however, reports also indicate that aggressive stone removal can be performed safely and may improve outcomes [8,9]. Therefore, to appropriately determine treatment strategies for patients with poor PS, a reliable predictive tool is needed that considers not only short-term mortality risk but also long-term prognosis.

Existing severity scores such as the sequential organ failure assessment (SOFA) and acute physiology and chronic health evaluation (APACHE) have been well validated for predicting short-term mortality in sepsis [10,11]. Some studies suggest that these scores may correlate with both mid-term and long-term outcomes; however, the evidence remains inconsistent, and no scoring system for predicting long-term prognosis has been established [12,13]. These tools require multiple clinical parameters, and the complexity of the system can be problematic [10,11,14]. They primarily reflect acute organ dysfunction rather than host reserve capacity or impaired homeostasis.

The FAN score is a composite score combining the fibrosis-4 (FIB-4) index, the albumin-bilirubin (ALBI) score, and the neutrophil-lymphocyte ratio (NLR), and has recently gained attention as a prognostic indicator in oncology [15]. Each component score reflects different aspects of host reserve capacity, such as liver function, systemic inflammation, and fibrotic burden. These factors are all closely associated with frailty and reduced physiological resilience [16] and have been reported as prognostic markers for sepsis patients, in addition to poor PS and malnutrition [1-4,17-23]. Given these characteristics, the FAN score was considered useful for evaluating the prognosis, particularly the mid-term and long-term prognosis, in older adult patients with sepsis and poor PS.

The primary objective of this study was to explore whether the FAN sepsis (FANs) score with modified cutoff values correlates with in-hospital (short-term), one-year (mid-term), and overall (long-term) mortality in patients with sepsis caused by obstructive pyelonephritis due to urinary tract stones. As a secondary objective, we compared the prognostic predictive ability of the FANs score and the SOFA score.

## Materials and methods

Data source and patient cohort

We identified 56 patients with a PS of 3 or 4 who were admitted to Kishiwada Tokushukai Hospital between April 2019 and March 2025 for sepsis or septic shock due to obstructive pyelonephritis caused by a urinary tract stone. Septic shock was defined using the Sepsis-3 criteria [24]. All drainage methods involved stent placement, and all patients who were not drained did not consent to active treatment. The treatment plan for UTI was left to the decision of the attending physician, the patient, and the patient's family. This retrospective study was conducted at a single center and approved by the Institutional Review Board of Kishiwada Tokushukai Hospital (approval number 25-16).

Data collection

Patient background data at admission, including age, gender, body mass index (BMI), Eastern cooperative oncology group (ECOG) PS (excluding death during hospitalization), Charlson comorbidity index (CCI), use of vasopressor, mean arterial pressure, lactic acid, laboratory data (procalcitonin, blood/urine cultures, urinary tract infection (UTI) characteristics (location, size, etc.), and SOFA score, were retrospectively recorded. All data were obtained before the start of treatment. Carbapenem was used as the initial antimicrobial agent and was changed to a susceptible antimicrobial agent, based on culture results, as appropriate. The FIB-4 index was calculated as follows [17]:

(Age [years] × AST [IU/L]) / (Platelet count [10⁹/L] × √ALT [IU/L])

The ALBI score was calculated as follows [18]:

(log₁₀ (T-bil [mg/dL] × 17.1) × 0.66) + (Albumin [g/dL] × 10 × (−0.085))

NLR was calculated as the neutrophil count divided by the lymphocyte count [19]. Cutoff values were defined based on previous studies in patients with sepsis: FIB-4 > 4.9, ALBI score > 1.39, and NLR > 10.42. Scores were calculated based on the original FAN score criteria [15]. Given the differing objectives and subjects, the cutoff values were modified, and the scoring system used in this study was defined as the FAN sepsis score (FANs score). All scoring systems used in this study (ECOG PS [5], SOFA score, FAN score, FIB-4 index, ALBI score, NLR, and CCI) are publicly available and have been previously described [5,10,15,17-19,25]. They are academically accessible.

Statistical analysis

Patients were classified according to the FANs score. The two groups were compared using the chi-square test or Fisher's exact test for categorical variables and the Kruskal-Wallis test for continuous variables. The Cochran-Armitage trend test was used to evaluate the association between the FANs score and mortality, and the 95% confidence interval was calculated using the percentile method with 2,500 bootstrap resamples. Kaplan-Meier and log-rank tests were used to estimate and compare the overall mortality rates between the groups based on the FANs score. Cox proportional hazards models were fitted for descriptive purposes; however, given the limited number of events, hazard ratio estimates may be unstable with extremely wide confidence intervals. ROC curves were constructed to evaluate the accuracy of prognosis prediction, and AUC and 95% confidence intervals were calculated. AUC comparisons were performed using the DeLong test. Given the exploratory nature and limited sample size, p-values were reported descriptively without adjustment for multiple testing. All statistical analyses were performed using the JMP Pro 18 software (SAS Institute Inc., Cary, NC, USA).

## Results

Patient characteristics

A total of fifty-six patients participated in this study, with results shown for the entire group and by the FANs score. (Table 1). The overall median age was 84 years (interquartile range [IQR], 76-83 years); 43 (76.7%) of the patients were female, and the median BMI was 20.5 kg/m2 (IQR, 17.7-22.4 kg/m2). ECOG PS was 3 and 4 in 39 (69.6%) and 17 (30.4%) patients, respectively. The CCI was 7 (IQR, 6-8), and vasopressors were used in 47 (83.9%) patients. Mean arterial pressure was 62.3 mmHg (IQR, 56.0-64.0 mmHg), lactate was 29 mg/dL (IQR, 19-51 mg/dL), and procalcitonin was 22.5 ng/mL (IQR, 7.1-50 ng/mL). More than half of the stones were located in the upper ureter. The median stone size was 8.5 mm (IQR, 7.0-10.5 mm), and drainage was performed in 26 (46.4%) patients. Ten patients (17.8%) had a FANs score of 0, 19 (33.9%) had a score of 1, 16 (28.6%) had a score of 2, and 11 (11.6%) had a score of 3. Although differences were observed in the use of vasopressors, mean arterial pressure, procalcitonin, platelet counts, C-reactive protein, serum creatinine, and SOFA scores (p=0.04, p=0.01, p<0.01, p<0.01, p=0.01, p<0.01, and p<0.01, respectively), there were no other significant differences between the two groups.

**Table 1:** Patient characteristics. All continuous variables are expressed as medians (IQR). ※1. Body mass index was calculated as weight (kg) divided by height squared (m²), according to the World Health Organization (WHO) definition. ※2. Serum lactate levels were expressed in mg/dL (1 mmol/L = 9.0 mg/dL). ※3. Excluding deaths during hospitalization. FAN sepsis score (FANs score): Based on the FAN Score [15], a composite index based on FIB-4 [17], ALBI [18], and NLR [19] with modified cutoff values from previous studies. ECOG PS: Eastern Cooperative Oncology Group Performance Status [5]. CCI: Charlson comorbidity index [25]. SOFA score: Sequential Organ Failure Assessment [10].

Characteristics	All (n=56)	FANs score 0 (n=10)	FANs score 1 (n=19)	FANs score 2 (n=16)	FANs score 3 (n=11)	p-value/ Test statistic
Age, year	84(76–88)	85.5(78.7–93.5)	83.0(76.0–86.0)	84.5(72.2–89.0)	86.0(70.0–88.0)	0.50/χ²= 2.35 (Kruskal–Wallis test)
Female, n (%)	43(76.7)	8(80.0)	15(78.9)	12(75.0)	8(72.7)	0.97/χ²= 0.23 (Fisher’s exact test)
Body mass index, kg/m2 ※1	20.5(17.7–22.4)	20.3(19.0–22.5)	21.2(18.0–21.5)	20.0(16.1–23.8)	18.4(17.7–23.6)	0.97/χ²= 0.19 (Kruskal–Wallis test)
ECOG PS, n (%)						0.27/χ²= 3.91 (Fisher’s exact test)
3	39(69.6)	8(80.0)	14(73.6)	12(75.0)	5(45.4)	
4	17(30.4)	2(20.0)	5(26.3)	4(25.0)	6(54.5)	
CCI	7(6–8)	7(6–7.25)	7(6–9)	6(5.0–7.7)	7(6–8)	0.71/χ²= 3.02 (Kruskal–Wallis test)
Use of vasopressor, n (%)	47(83.9)	6(60.0)	15(78.9)	16(100.0)	10(90.9)	0.04zχ²= 8.05 (Fisher’s exact test)
Mean arterial pressure, mmHg	62.3(56.0–64.0)	64.5(61.8.3–65.0)	63(60.0–63.7)	60.6(53.3–63.2)	58.3(53.0–62.3)	0.01/χ²= 10.36 (Kruskal–Wallis test)
Lactate, mg/dL※2	29(19–51)	29(21.0–35.2)	20.0(18.0–37.8)	52.5(20.5–68.75)	29.0(18.0–45.0)	0.06/χ²= 7.32 (Kruskal–Wallis test)
Procalcitonin, ng/mL	22.5(7.1–50)	3.0(0.11–22.2)	12.7(0.58–27.0)	50.0(20.6–71.7)	35.2(13.9–50)	<0.01/χ²= 18.85 (Kruskal–Wallis test)
Leukocyte counts, 103/μL	11.8(6.5–16.2)	6.0(2.5–13.4)	12.2(6.6–16.9)	12.7(8.6–21.1)	11.6(7.7–33.4)	0.24/χ²= 4.13 (Kruskal–Wallis test)
Platelet counts, 104/μL	12.9(8.0–18.4)	19.8(13.3–22.7)	15.1(11.8–20.9)	10.4(5.9–13.4)	6.7(4.2–9.2)	<0.01/χ²= 16.13 (Kruskal–Wallis test)
C-reactive protein, mg/dL	11.9(4.6–19.4)	5.8(1.4–13.0)	8.8(3.7–12.0)	16.5(6.9–25.8)	15.5(10.6–22.7)	0.01/χ²= 10.62 (Kruskal–Wallis test)
Serum creatinine, mg/dL	1.6(1.0–2.6)	1.0(0.8–1.4)	1.2(0.8–1.82)	2.1(1.3–3.6)	2.0(1.7–4.5)	<0.01/χ²= 16.53 (Kruskal–Wallis test)
Infections of multidrug-resistant bacteria, n(%)	16(28.5)	2(20.0)	5(26.3)	4(25.0)	5(45.4)	0.56/χ²= 2.04 (Fisher’s exact test)
Laterality of stone, n (%)						0.09/χ²= 6.35 (chi-square test)
Right	27(51.7)	6(60.0)	12(63.1)	9(56.2)	2(18.1)	
Left	29(48.3)	4(40.0)	7(36.8)	7(43.7)	9(81.8)	
Position of stone, n (%)						0.62/χ²= 7.15 (Fisher’s exact test)
Pelvic ureteral junction and Upper ureter	38(67.8)	7(70.0)	11(57.8)	11(68.7)	9(81.8)	
Mid ureter	1(1.8)	0(0)	1(5.2)	0(0)	0(0)	
Lower ureter	17(30.4)	3(30.0)	7(36.8)	5(31.2)	2(18.1)	
Stone size, mm	8.5(7.0–10.5)	8.3(5.6–9.9)	10.0(7.35–12.8)	8.0(6.2–9.6)	8.5(7.4–12.2)	0.15/χ²= 5.31 (Kruskal–Wallis test)
Drainage, n (%)	30(53.3)	5(50.0)	8(42.1)	9(56.2)	7(63.6)	0.56/χ²= 2.01 (chi-square test)
Post-treatment, n(%)※3						0.70/χ²= 9.02 (Fisher’s exact test)
Progress observation	24(42.8)	5(50.0)	8(42.1)	7(43.7)	4(36.3)	
Routine stent replacement	3(5.3)	0(0)	1(5.2)	1(6.2)	1(9..0)	
Extracorporeal shock wave lithotripsy	5(8.9)	2(20.0)	2(10.5)	1(6.2)	0(0)	
Transurethral lithotomy	15(26.7)	3(30.0)	6(31.5)	4(25.0)	2(18.1)	
Stone free, n(%)	30(53.5)	7(70.0)	10(52.6)	8(50.0)	5(53.9)	0.69/χ²= 1.46 (chi-square test)
SOFA score, n (%)	9(6.0–11.0)	6(3.0–8.25)	8(5.0–9.0)	10(8.2–13.0)	11(10.0–13.0)	<0.01/χ²= 18.84 (Kruskal–Wallis test)

Association Between the FANs Score and In-Hospital Mortality Rate, One-Year Mortality Rate, and Overall Mortality Rate

**Figure 1:**
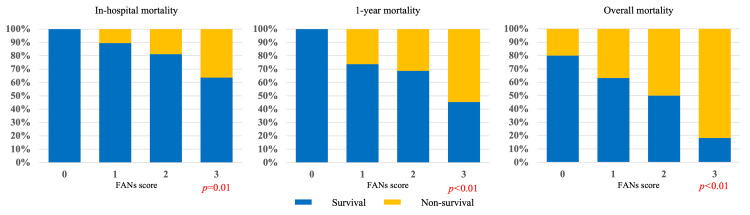
Cochran-Armitage trend test for the association between FANs scores and in-hospital, one-year, and overall mortality. FAN sepsis score (FANs score): Based on the FAN score [15], a composite index based on FIB-4 [17], ALBI [18], and NLR [19] with modified cutoff values from previous studies.

In-hospital mortality, one-year mortality, and overall mortality increased progressively with rising FANs scores (Table 2, Figure 1). Higher FANs scores were associated with increased mortality and a trend toward lower survival rates. In contrast to patients with FANs score 0, patients with FANs score 3 had the highest risk, with in-hospital mortality of 36.3% (95% CI 18.1-72.7), one-year mortality of 54.5% (95% CI 36.3-90.9), and overall mortality of 81.8% (95% CI 63.6-100). The differences across FANs groups were statistically significant for all outcomes (in-hospital p=0.01; one-year and overall mortality both p<0.01). These findings indicate that higher FANs scores were consistently associated with increased in-hospital, one-year, and overall mortality.

**Table 2:** Mortality outcomes stratified by FANs score groups, showing stepwise increases in in-hospital, one-year, and overall mortality. FAN sepsis score(FANs score): Based on the FAN score [15], a composite index based on FIB-4 [17], ALBI [18], and NLR [19] with modified cutoff values from previous studies.

FANs score (n=56)	In-hospital mortality, % (95% CI）	1-year mortality, %(95% CI)	Overall mortality, %(95% CI)
0(n=10)	0(NA-NA)	0(NA-NA)	20.0(10.0-50.0)
1(n=19)	10.5(5.2-32.9)	26.3(10.5-52.6)	36.8(21.0-63.1)
2(n=16)	18.7(6.2-47.9)	31.2(18.7-68.7)	50.0(31.2-81.2)
3(n=11)	36.3(18.1-72.7)	54.5(36.3-90.9)	81.8(63.6-100)

Association Between the FANs Score and Overall Mortality

**Figure 2:**
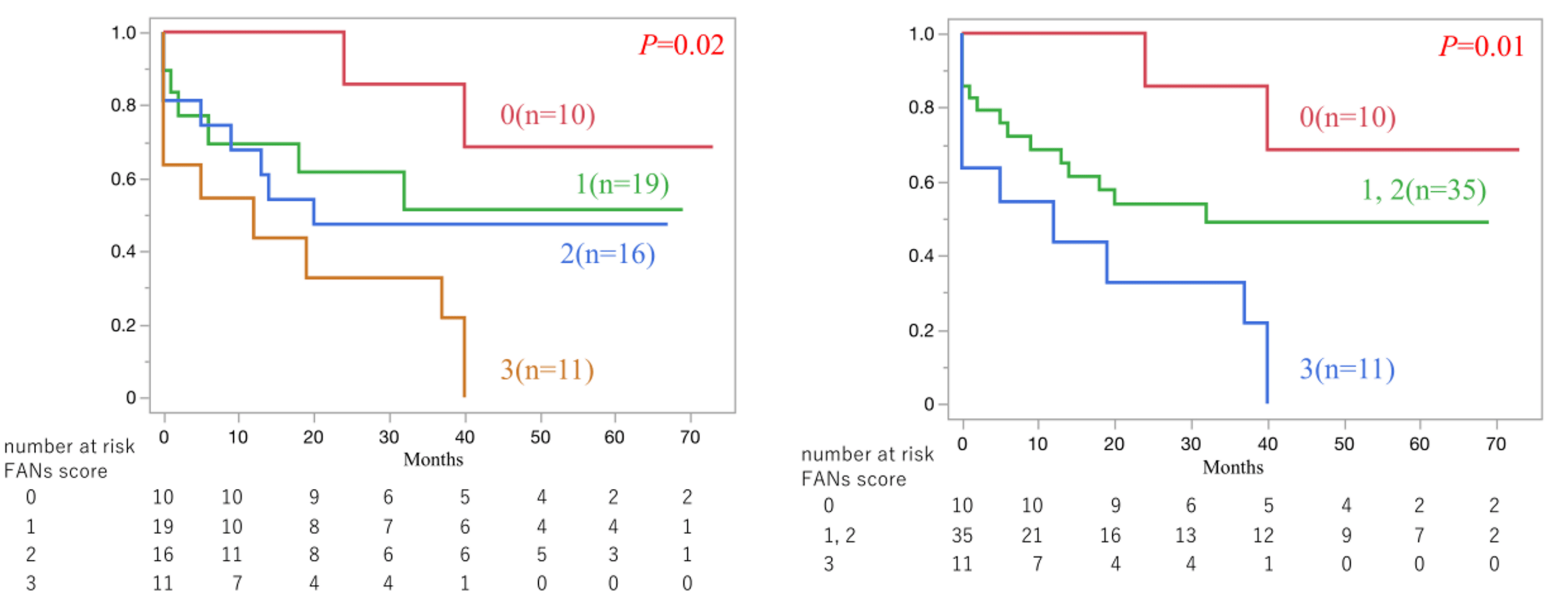
Kaplan–Meier analysis of overall survival by FANs score (2a) and by risk (2b). Score 0 is defined as low risk, 1 and 2 as intermediate risk, and 3 as high risk. FAN sepsis score (FANs score): Based on the FAN Score [15], a composite index based on FIB-4 [17], ALBI [18], and NLR [19] with modified cutoff values from previous studies.

We assessed overall mortality, which was analyzed as overall survival (OS) using Kaplan-Meier curves. The median follow-up was 18.5 months (IQR, 1-40.7 months), during which 26 patients died. Seventeen patients died during hospitalization, of which four (28.5%) were UTI-related. As shown in Figure 2a, there was a significant difference in OS between patients with different FANs scores (p=0.02). Since there was no significant difference between scores of 1 and 2 (p=0.71), they were considered medium risk, with 0 indicating low risk and 3 indicating high risk. As shown in Figure 2b, there was a significant difference in OS between the different risk groups (p=0.01). The unadjusted hazard ratio for the comparison between low-risk and high-risk was 6.6 (p=0.01), but the 95% confidence interval was wide (1.4-31.3), reflecting instability of the estimate due to a small number of events and clustered deaths. Therefore, this HR should be interpreted as a descriptive indication of direction rather than a precise estimate; we have not tabulated such unstable CIs. Furthermore, the comparison between the low-risk and medium-risk groups did not reach statistical significance, likely due to the small sample size.

Comparison of the Predictive Ability of FANs Scores and SOFA Scores for In-Hospital Mortality, One-Year Mortality, and Overall Mortality

**Figure 3:**
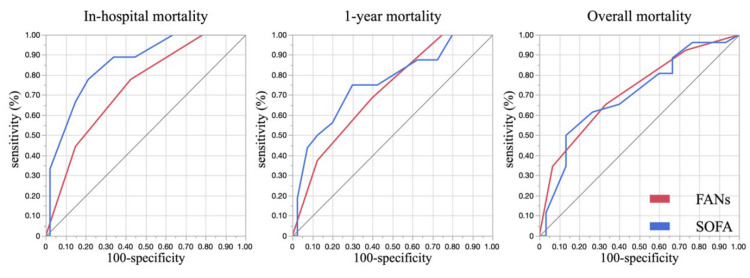
Receiver operating characteristic (ROC) curves comparing FANs and SOFA scores for prediction of in-hospital (short-term), one-year (mid-term), and overall (long-term) mortality in patients with sepsis due to obstructive pyelonephritis. FAN sepsis score (FANs score): Based on the FAN Score [15], a composite index based on FIB-4 [17], ALBI [18], and NLR [19] with modified cutoff values from previous studies. SOFA score: Sequential Organ Failure Assessment [10].

Receiver operating characteristic (ROC) curves for in-hospital, one-year, and overall mortality are shown in Figure 3. The AUC values of the FANs score were 0.74, 0.71, and 0.72, respectively. The corresponding AUCs of the SOFA score were 0.83, 0.75, and 0.70. AUC comparison was performed using the DeLong test for correlated ROC curves. Although SOFA tended to perform better for in-hospital mortality, there were no statistically significant differences in AUCs between the two scores across all time points (p=0.17, p=0.50, p=0.80, respectively).

## Discussion

In patients with frailty, like poor PS, aggressive treatment may not contribute to the prognosis due to poor homeostasis, which essentially refers to physiological reserve and systemic resilience, and a limited life expectancy [16]. Therefore, the treatment and patient selection are important issues. In this exploratory study of patients with sepsis due to obstructive pyelonephritis, higher FANs scores were associated with increased in-hospital, one-year, and overall mortality. While SOFA demonstrated superior discriminatory ability for in-hospital mortality, no significant difference was observed. FANs and SOFA showed equivalent predictive capabilities for one-year and overall mortality. Although other biomarkers such as the FIB-4 index, ALBI score, and NLR have been reported to be associated with short-term prognosis in patients with sepsis [17-19], we believe that these markers will serve as indicators of both short- and long-term prognosis. To our knowledge, this is the first report to demonstrate an association between FANs score and prognosis in patients with septic shock. Unlike existing scores, this score may predict prognosis based solely on blood sampling without requiring clinical evaluation. We believe it will be highly useful in selecting treatment options for frail patients with poor performance status.

The FIB-4 index was initially developed to assess liver fibrosis in patients with viral hepatitis [17]. However, it reflects not only liver fibrosis but also the degree of systemic inflammation and its impact on multiple organ systems, including the liver. It has been shown to correlate with sepsis severity and prognosis [17]. A cutoff value of 4.9 was significantly associated with increased 28-day mortality (p<0.01). Similarly, the ALBI score has been applied to the prognostic evaluation of patients with hepatocellular carcinoma and liver injury [26,27]. A cutoff value of -1.39 is indicative of severe hepatic dysfunction, and mortality increases significantly with increasing grades of the ALBI score [18]. They are not only indicators of liver function and prognosis in patients with sepsis but are also associated with prognosis in patients with cancer and coronavirus disease [15,28]. The liver plays a pivotal role in regulating proteins and immune responses. Frailty decreases the regenerative capacity of the liver and causes inflammation, thereby worsening the prognosis [23]. Patients with frailty and poor PS are more likely to develop liver damage due to inflammation, and the degree of liver damage in sepsis is thought to be related to the prognosis.

The NLR has also been studied as an easier way to diagnose sepsis and shows high values, reflecting the effects of inflammation [29]. A cutoff value of 10.42 significantly correlates with prognosis in patients with sepsis (p<0.01) and is considered a prognostic marker comparable to the SOFA score [19].

Kawashima et al. established the FAN score using data from patients with metastatic urothelial carcinoma who were treated with pembrolizumab. The FAN score was calculated using the FIB-4 index, ALBI score, and NLR and was correlated with prognosis in patients with cancer [15]. Because it is difficult to use the FAN score directly to predict the prognosis of patients with sepsis, it was modified using cutoff values from previous reports and used as the FANs score. Recently, the FAN scoring system was reported to be useful in patients with heart failure [30]. This suggests that it may be useful in different fields. Indeed, we hypothesized that the association between the degree of inflammation and the prognosis of disability in patients with poor PS would be partially similar, although the backgrounds of patients with cancer and those with sepsis are different. Supporting this idea, the present study indicated that FANs scores were associated not only with in-hospital but also with one-year to overall mortality.

In the present study, the FANs score (AUC=0.72) and SOFA score (AUC=0.70) have comparable prognostic ability (p=0.80). This indicates moderate predictive accuracy. An AUC above 0.7 suggests that the score can reasonably discriminate patients at higher risk of overall mortality, supporting its potential clinical utility. The present results extend this literature by suggesting that a laboratory-based score, such as the FANs score, may capture long-term vulnerability in septic patients with poor PS. The FANs score requires only laboratory parameters and may therefore provide a simple and feasible tool for prognostic assessment.

This study has several limitations. First, because of the small sample size and single-center, retrospective design, the findings should be considered exploratory rather than confirmatory. We did not divide the cohort into training and validation sets, and external validation was not feasible. Future studies with larger, prospectively collected datasets are warranted to validate the prognostic performance of the FANs score. Second, we used modified cutoff values for each component of the FANs score based on distributions and previous evidence in septic populations. These cutoffs were intended to adapt the original FAN score to the characteristics of sepsis patients but require independent confirmation. Third, frailty was discussed as a conceptual framework because all patients had poor performance status (ECOG PS 3-4), but no formal frailty scales, such as the Clinical Frailty Scale, were applied. Incorporating objective frailty assessments in future research would strengthen the analysis. Finally, the small number of events may have led to unstable hazard ratio estimates. Therefore, all inferential results should be interpreted descriptively. Given these methodological limitations, the present study should be regarded as hypothesis-generating. The findings support the potential clinical relevance of the FANs score, but further prospective validation in larger, independent cohorts is essential before clinical application. Despite these limitations, we believe that our findings provide meaningful preliminary insights into the potential prognostic value of the FAN score in septic patients.

## Conclusions

These results suggest that a high FANs score may be a poor prognostic factor in patients with frailty, such as a poor performance status, who develop septic shock due to urinary tract obstruction. Patients with higher FANs scores tended to have worse short- and long-term survival outcomes compared with those with lower scores. This finding may be clinically useful for predicting prognosis and guiding management decisions in frail patients with poor PS and sepsis.
